# Clonal Expansion of the Macrolide Resistant ST386 within Pneumococcal Serotype 6C in France

**DOI:** 10.1371/journal.pone.0090935

**Published:** 2014-03-06

**Authors:** Claire Janoir, Robert Cohen, Corinne Levy, Edouard Bingen, Agnès Lepoutre, Laurent Gutmann, Emmanuelle Varon, the Observatoires Régionaux du Pneumocoque (ORP) network

**Affiliations:** 1 Centre National de Référence des Pneumocoques (CNRP), AP-HP Hôpital Européen Georges-Pompidou, Paris, France; 2 Département de Microbiologie, Faculté de Pharmacie EA4043, Châtenay-Malabry, France; 3 Département de Microbiologie, Centre Hospitalier Intercommunal de Créteil, Créteil, France; 4 Centre de Recherche Clinique, Centre Hospitalier Intercommunal de Créteil, Créteil, France; 5 Association Clinique Thérapeutique Infantile du Val de Marne (ACTIV), Saint-Maur-des-Fossés, France; 6 Groupe de Pathologie Infectieuse Pédiatrique (GPIP), Paris, France; 7 Département de Microbiologie, Université Denis-Diderot-Paris 7, AP-HP Hôpital Robert-Debré, Paris, France; 8 Département de Maladies Infectieuses, Institut de Veille Sanitaire, Saint-Maurice, France; 9 Laboratoire de Microbiologie, AP-HP Hôpital Européen Georges-Pompidou, Paris, France; 10 INSERM, U872, Paris, France; Rockefeller University, United States of America

## Abstract

In France, the use of the 7-valent pneumococcal conjugate vaccine (PCV7) lead to an overall significant decrease in PCV7 invasive pneumococcal disease (IPD) incidence. However, the decrease in vaccine serotype prevalence was partially counterbalanced by the serotype replacement phenomenon. In this study, we analyzed the role of the newly described serotype 6C as one of the replacement serotypes. This work was conducted on a large time scale from the early PCV7 era (2002–2003) to the PCV13 era (2010–2011), both on IPD strains recovered from the whole population and nasopharyngeal colonizing strains isolated in infant less than two years, who are known to be the main reservoir for pneumococci. Serotype 6C took advantage over 6A and 6B serotypes, which both decreased over time. A continuous and significant increase in 6C IPD was observed in adults along the study period; in contrast, in children less than two years, only an increase in 6C nasopharyngeal carriage was found, the prevalence of serotype 6C in IPD remaining very low over time. Among 101 6C invasive and colonizing strains studied by MLST, 24 STs were found to be related to three major clonal complexes, CC395, CC176, and CC315. STs related to CC176 tend to disappear after 2009 and were essentially replaced by ST386 (CC315), which dramatically increased over time. This clonal expansion may be explained by the erythromycin and tetracycline resistances associated with this clone. Finally, the decrease observed in nasopharyngeal 6C carriage since 2010, likely related to the PCV13 introduction in the French immunization schedule, is expected to lead to a decrease in 6C IPD in adults thereafter.

## Introduction

*Streptococcus pneumoniae* is an important human pathogen, which can cause various diseases, ranging from mild otitis media to life-threatening diseases such as severe pneumonia, bacteremia and meningitis. It has been considered to be responsible for more than one million deaths annually worldwide, mainly among young children and the elderly [1]. However, pneumococcus is also a common commensal of the nasopharynx, mostly in children, which constitutes its natural main reservoir. The major virulence factor is the polysaccharide capsule, which shields pneumococci from host phagocytes [2]. *S. pneumoniae* expresses at least 92 different immunogenic capsules, but 75% of invasive pneumococcal diseases (IPD) are caused by about a dozen of serogroups alone. Before the introduction of the 7-valent pneumococcal conjugate vaccine (PCV7), the “historical” serogroup 6, which included serotypes 6A and 6B, was among the five prominent serogroups recovered from IPD on each continent [3]. The capsular polysaccharides produced by serotype 6A and 6B strains are very similar: both have repeating units composed of galactose-glucose-rhamnose-ribitol-phosphate, with a linkage between rhamnose and ribitol, which is of the 1→3 type for 6A and of the 1→4 type for 6B. This difference is correlated with point mutations in the *wciP* gene encoding the putative rhamnosyltransferase, leading to amino acid substitution at positions 195 [4], 192 and 254 [5].

In recent years, two new serotypes C and D belonging to serogroup 6 have been discovered. The serotype 6C, which is serologically very similar to serotype 6A, was identified using monoclonal antibody [6]. It was initially thought to derive from serotype 6A following replacement of the *wciN-*6A gene encoding a galactosyltransferase by the *wciN*-6C gene encoding a glycosyltransferase [7]. This resulted in a single substitution of a galactose with a glucose unit [7], [8]. Subsequently, it was suggested that a larger genetic fragment from unknown origin and spanning *wciN* and *wzy* was recombined in pneumococcal genetic backgrounds [9]. Serotype 6D displayed the same galactosyltransferase*wci*N-6C gene, but in a 6B genetic background [10].

Serotype 6B and 19F have been included in the 23-valent purified capsular polysaccharide vaccine, which is recommended in at risk population more than 2 years old, and in the conjugate vaccines (PCV7 and PCV13) recommended for children under 5 years. These vaccines provide serotype-specific protection, however it was hoped that protection conferred by the first licensed PCV7 would cover, at least partially, the closely related serotypes not included in PCV7, namely 6A and 19A. By contrast to serotype-specific 19F antibodies which were unable to induce cross protection against serotype 19A [11], an *in vitro* opsonisation study using serotype-specific 6B antibodies showed a cross protection for the 6A serotype, but no cross protection for the 6C serotype [12]. This was consistent with studies which documented, since the introduction of the PCV7, an increase in serotype 6C IPD or carriage in several countries worldwide [13]–[18]. The PCV7 was recommended in France on March 2002, for children at risk for pneumococcal disease, but the vaccine coverage stayed quite low (<50%) until 2006, when it was generalized for all children less than two years. Then it increased slowly to reach 59% in 2007, 78% in 2008, and 85% in 2010 [19]. Since June 2010, the 13-valent pneumococcal conjugate vaccine (PCV13) replaced the PCV7 in France and its coverage reached 88% in 2011 (http://www.invs.sante.fr/fr/Dossiers-thematiques/Maladies-infectieuses/Maladies-a-prevention-vaccinale/Couverture-vaccinale/Donnees/Pneumocoque). This new vaccine formulation includes the serotype 6A conjugate in addition to the 6B, but not the 6C.

In this study, we investigated the changes in epidemiology of the serogroup 6 strains in France, from the early PCV7 era 2002–2005 to the PCV13 era 2010–2011, focusing on prevalence, susceptibility profile, and multilocus sequence typing (MLST) of the newly recognized serotype 6C isolates. Given the role of the natural reservoir that constitutes the nasopharynx of young children in the transmission of pneumococci, we take the opportunity of the unique French on-going survey of pneumococcal carriage in children less than two years, which was initiated in 2001, to compare trends observed on one hand, on invasive strains from meningitis and bacteremia and, on the other hand, on nasopharynx colonizing strains.

## Materials and Methods

### Ethics Statement

The protocol of the carriage study was approved by the Saint Germain en Laye Hospital Ethics Committee, and written informed consent was obtained from the parents or guardians.

### *Streptococcus pneumoniae* Isolates

A total of 12,345 pneumococci isolated from blood cultures (8,527 strains) or cerebrospinal fluid (CSF, 3,818 strains), from children (n = 4,221) or adults (n = 8,124), were studied at the National Reference Center for Pneumococcus (CNRP). These strains were collected as part of the French national survey program of pneumococcal infections, through the “Observatoires Régionaux du Pneumocoque”, a network of 400 laboratories located in the 22 regions of France and covering at least 70% of the admissions in medical wards. Since 2001, CNRP received all invasive isolates from children (<16 years old), all meningitis and 20% of bacteremia isolates from adults.

A total of 4,637 nasopharyngeal (NP) colonizing isolates collected during the same period was studied. These isolates were recovered by nasopharyngeal swabs in children aged 6 to 23 months, suffering from acute otitis media, as part of a national surveillance study on *S. pneumoniae* nasopharyngeal carriage. Samples were collected by pediatricians throughout the year except for July and August [20].

The repartition of the corresponding invasive and colonizing serogroup 6 isolates throughout time and according to age is presented in Tables 1 and 2.

**Table 1:** Distribution of serotypes 6A, 6B, and 6C among *Streptococcus pneumoniae* isolates recovered from invasive pneumococcal disease (meningitis and bacteremia, n = 12,345) according to age group, from 2002 to 2011.

		Early PCV7 era		Late PCV7 era		PCV13 era	Trend
No of Isolates (%)	Age group	2002–2003	2004–2005	2006–2007	2008–2009	2010–2011	*p* *
**All serotypes**	All ages	2581	2015	2341	2833	2575	
	0–23 months	434	376	398	483	359	
	2–15 years	343	425	424	500	455	
	16–64 years	892	535	742	970	933	
	>64 years	878	444	777	880	828	
	Unknown	34	235	0	0	0	
**Serogroup 6**	All ages	281 (10.9)	195 (9.7)	153 (6.5)	123 (4.3)	135 (5.2)	Decrease <10^−4^
**Serotype 6B**	All ages	199 (7.7)	111 (5.2)	72 (3.1)	27 (1.0)	12 (0.5)	Decrease <10^−4^
	0–23 months	71 (16.4)	33 (8.8)	12 (3.0)	6 (1.2)	1 (0.3)	<10^−4^
	2–15 years	18 (5.2)	20 (4.7)	18 (4.2)	1 (0.2)	1 (0.2)	<10^−4^
	16–64 years	44 (4.9)	27 (5.0)	24 (3.2)	7 (0.7)	6 (0.6)	<10^−4^
	>64 years	66 (7.5)	21 (4.7)	18 (2.3)	13 (1.5)	4 (0.5)	<10^−4^
	Unknown	0	10	0	0	0	NA
**Serotype 6A**	All ages	55 (2.1)	56 (2.8)	57 (2.4)	37 (1.3%)	32 (1.2)	Decrease <10^−3^
	0–23 months	16 (3.7)	11 (2.9)	12 (3.1)	5 (1.0)	3 (0.8)	<0.05
	2–15 years	3 (0.9)	14 (3.3)	4 (0.9)	1 (0.2)	1 (0.2)	<0.05
	16–64 years	15 (1.7)	11 (2.1)	19 (2.5)	15 (1.5)	15 (1.6)	NS
	>64 years	21 (2.4)	16 (3.6)	22 (2.8)	16 (1.8)	13 (1.6)	NS
	Unknown	0	4	0	0	0	NA
**Serotype 6C**	All ages	16 (0.6)	16 (0.8)	22 (0.9)	59 (2.1)	91 (3.5)	Increase <10^−4^
	0–23 months	1 (0.2)	0	1 (0.3)	2 (0.4)	3 (0.8)	NS
	2–15 years	0	2 (0.5)	– (0)	3 (0.6)	7 (1.5)	<0.05
	16–64 years	10 (1.1)	6 (1.1)	11 (1.5)	26 (2.7)	43 (4.6)	<10^−4^
	>64 years	5 (0.6)	5 (1.1)	10 (1.3)	28 (3.2)	38 (4.6)	<10^−4^
	Unknown	0	3	0	0	0	NA
**Serotype 6A/C**°	All ages	11	12	2	0	0	NA

Introduction of PCV7 in France, March 2002, for children at high risk for IPD; Generalization of PCV7 for children <2 years in 2006; Introduction of PCV13 in France, June 2010.

*Significant trend over the 5 periods, calculation was performed by the extended Mantel-Haenszel Chi square test for linear trend.

°Serotype conventionally determined as 6A that could not be further tested for serotype 6C.

NA, Not applicable; NS, not significant.

**Table 2:** Distribution of serotypes 6A, 6B and 6C among *Streptococcus pneumoniae* isolates recovered from nasopharyngeal carriage in children aged 6–23 months, from 2002 to 2011.

No of Isolates (%)	Whole study period	Early PCV7 era		Late PCV7 era		PCV13 era	Trend
		2002–2003	2004–2005	2006–2007	2008–2009	2010–2011	p*
**All serotypes**	4,637	618	895	863	1056	1205	NA
**Serogroup 6**	550 (11.9)	124 (20.1)	136 (15.2)	92 (10.7)	85 (8.0)	113 (9.4)	Decrease <10^−4^
**Serotype 6B**	170 (3.7)	71 (11.5)	56 (6.3)	21 (2.4)	11 (1.0)	11 (0.9)	Decrease <10^−4^
**Serotype 6A**	186 (4.0)	40 (6.5)	58 (6.5)	46 (5.3)	28 (2.7)	14 (1.2)	Decrease <10^−4^
**Serotype 6C**	192 (4.1)	12 (1.9)	22 (2.5)	25 (2.9)	45 (4.3)	88 (7.3)	Increase <10^−4^
**Serotype 6A/C**°	2	1	–	–	1	–	NA

Introduction of PCV7 in France, March 2002, for children at high risk for IPD; Generalization of PCV7 for children <2 years in 2006; Introduction of PCV13 in France, June 2010.

*Significant trend over the 5 periods, calculation was performed by the extended Mantel-Haenszel Chi square test for linear trend.

°Serotype conventionally determined as 6A that could not be further tested for serotype 6C.

NA, Not applicable.

### Serotyping

All isolates were serotyped using latex particles sensitized with antisera from the Statens Serum Institute (Copenhagen, Denmark). Before 2010, no serotype 6C specific antiserum was available, and thus all 6A and 6C isolates have been conventionally typed as 6A (CS6A). Since 2010, a new specific-6C factor serum named “6d” was marketed by the Statens Serum Institute [21], also with a new 6A-specific factor serum named “6b*”; since then, both serotypes 6A and 6C were properly identified using these new factor sera. Validation of this serotyping reaction as compared to the PCR-serotyping method has been done elsewhere [22], and was performed also in this study for 12 strains randomly chosen. The 6C reference strain ahoy702, a kind gift from Peter Hermans, was used as internal quality control.

### Serotype 6C Determination by PCR

Among the CS6A pneumococci isolated from 2002 to 2009, further identification of serotype 6C was retrospectively assessed by PCR amplification of the *wciN* genes. Using the forward primer 5106 and the reverse primer 3101 as previously described [7], [15], serotype 6A and 6C isolates displayed a 2.0 kb and a 1.8 kb amplified fragment, respectively. Therefore, assessment of serotype was performed by evaluation of amplified fragment length in 2% agarose gel.

### Susceptibility Testing

MICs of penicillin, amoxicillin, and cefotaxime were performed by the agar dilution method (CA-SFM), and for other antibiotics (erythromycin, tetracycline), susceptibility testing was performed using the agar diffusion method. Results were interpreted according to the 2011 EUCAST guidelines (http://www.eucast.org/clinical_breakpoints/). Isolates were classified as penicillin-susceptible (MIC≤0.06 mg/L), penicillin non-susceptible (PNSP) (MIC>0.06 mg/L), penicillin intermediate-resistant (0.06<MIC≤2 mg/L), or penicillin resistant (PRP) (MIC>2 mg/L). *S. pneumoniae* ATCC49619 was used as internal quality control.

### MLST Analysis

Multilocus sequence typing (MLST) analysis was carried out on 101 serotypes 6C isolates (71 invasive and 30 carriage isolates), randomly taken through each of the periods. Internal fragments of the house-keeping genes *aroE*, *gdh*, *gki*, *recP*, *spi*, *xpt* and *ddl* were amplified by PCR using protocol previously described [23], except that some primers were modified as following: *aroE*-up2, 5′-TGATGGCTATACACGTTTAGC-3′; *aroE*-dn2, 5′-ATTGCCCTGACTTCTAGC-3′; *recP*-dn2, 5′-TGCATAGCAGCATGGATGG-3′; *spi*-up2, 5′-AACGCTTAGAAAGGTAAGT-3′; *xpt*-up2, 5′-TTTAGACTTTAGGAGGTC-3′; and *ddl*-dn2, 5′-CGCTCGATTAGTTCTGGGTA-3′. Allele and sequence-type (ST) assignments were made using the MLST Web site (available at http://spneumoniae.mlst.net/). STs were grouped on the basis of five out of seven identical alleles, according to eBURST-v3 analysis of the STs obtained for the 101 isolates of this study in addition to the global database, and designated by their respective predicted founder. New alleles and STs were submitted to the MLST database curator.

### Data Analysis

Data recovered between 2002 and 2011 were analyzed by two-years periods, the 2002–2003 and 2004–2005 periods corresponding to the early PCV7 era (PCV7 was recommended in March 2002), the 2006–2007 and 2008–2009 periods to the late PCV7 period, during which the vaccine coverage exceeded 50% of the target population once generalization of the vaccine was allowed, and the PCV13 era. At this latter period, PCV13 replaced PCV7 in June 2010. Analysis of 6A, 6B, and 6C specific IPD cases were compared to the total number of IPD isolates by each age group. Furthermore, a refined analysis was done, comparing the last year of PCV7 exclusive use (2009), the year of transition (2010) and the first complete year of PCV13 exclusive use (2011), in order to evaluate any change associated to the PCV13 introduction. Statistical analyzes on the serotypes distribution over time were performed using the Open Source Epidemiologic Statistics for Public Health (http://www.openepi.com/menu/openEpiMenu.htm). Significant trend test over the five periods were performed by the extended Mantel-Haenszel Chi square test for linear trend.

## Results

### Distribution of Serotype 6A, 6B, and 6C Isolates in IPD and NP Carriage, from 2002 to 2011 in France

Among the 12,345 IPD isolates studied at the CNRP between 2002 and 2011 (Table 1), 887 belonged to group 6, 421 were identified as serotype 6B, and 466 were initially conventionally serotyped as 6A (CS6A). Among 441 CS6A strains available for further analysis, 237 were confirmed as true 6A, while 204 were ultimately identified as 6C. Opposite trends in prevalence were observed for serotype 6A or 6B isolates on one hand, and for serotype 6C on the other hand (Table 1). The prevalence of 6B isolates dramatically decreased from 7.7% (199/2581) in the early PCV7 era 2002–2003 to 0.5% (12/2575) in the PCV13 era 2010–2011 (p<10^−4^), a trend observed in all age groups (Table 1). Regarding 6A isolates, a significant overall decrease was observed from 2002–2003 (2.1%) to 2010–2011 (1.2%) (p<10^−3^). However, a significant decrease in 6A prevalence was exclusively observed in children (<16 years) but not in the rest of the population where it remained stable. This was in contrast to an overall gradual increase in the newly recognized serotype 6C from 0.6% in 2002–2003 to 3.5% in 2010–2011 (p<10^−4^). Prevalence of serotype 6C remained very low in children less than 2 years, increased moderately for those between 5 and 15 years, and the widest increment was observed in the adults over 15 years. Thus, in children less than 2 years, the significant decrease in the prevalence of serotype 6A and 6B IPD isolates was not balanced by an increase in serotype 6C IPD isolates, while in adults, the decrease of 6A and 6B IPD isolates was completely offset by the increase in 6C. Of note, after several years of continuous although moderate increase, a stabilization of the 6C prevalence was observed between 2010 and 2011 in this latter population (Table 3), following the introduction of PCV13 in June 2010 for children less than 2 years.

**Table 3:** Prevalence of serotype 6C isolates from 2009 to 2011, in nasopharyngeal (NP) carriage and in invasive pneumococcal disease (IPD) according to age.

No of isolates (%)	Age group	Serotype	2009	2010	2011	2009/2010	2010/2011
						p	p
**IPD**	0–23 months	All	278	188	171		
		6C	1 (0.3)	1 (0.5)	2 (1.2)	NS*	NS*
	2–15 years	All	315	198	257		
		6C	3 (1.0)	2 (1.0)	5 (1.9)	NS*	NS*
	>15 years	All	1064	758	1003		
		6C	38 (3.6)	41 (5.4)	40 (4.0)	0.03°	NS°
**NP carriage**	6–23 months	All	509	503	702		
		6C	26 (5.1)	50 (9.9)	38 (5.4)	<0.002°	<0.003°

*Calculation was performed by the Fisher exact test.

°Calculation was performed by the Mantel-Haenszel Chi square test.

NS, Not significant.

During the same period, 550 of 4,637 pneumococcal colonizing isolates recovered from the nasopharynx of children less than two years during acute otitis media were found to belong to serogroup 6 (Table 2). Parallel to the trend observed in IPD among children less than 2 years, the prevalence of 6B isolates in NP carriage decreased more than 10-fold from 11.5% in 2002–2003 to 0.9% in 2010–2011 (p<10^−4^). Out of 380 CS6A isolates, 378 were further analyzed. If the distribution of the CS6A isolates looks stable over time, the proportion of “true” 6A isolates gradually declined from 6.5% in 2002–2003 to 1.2% in 2010–2011 (p<10^−4^). In contrast, the overall proportion of 6C isolates significantly increased from 1.9% in 2002–2003 to 7.3% in 2010–2011 (p<10^−4^). A refined analysis of NP carriage isolates during the three last years of the study period showed a significant increase in serotype 6C colonizing isolates between 2009 and 2010 (5.1% vs. 9.9%, p<0.002), followed by a significant decline in 2011 (5.4%) (p<0.005) (Table 3).

### MLST Analysis

**Figure 1:**
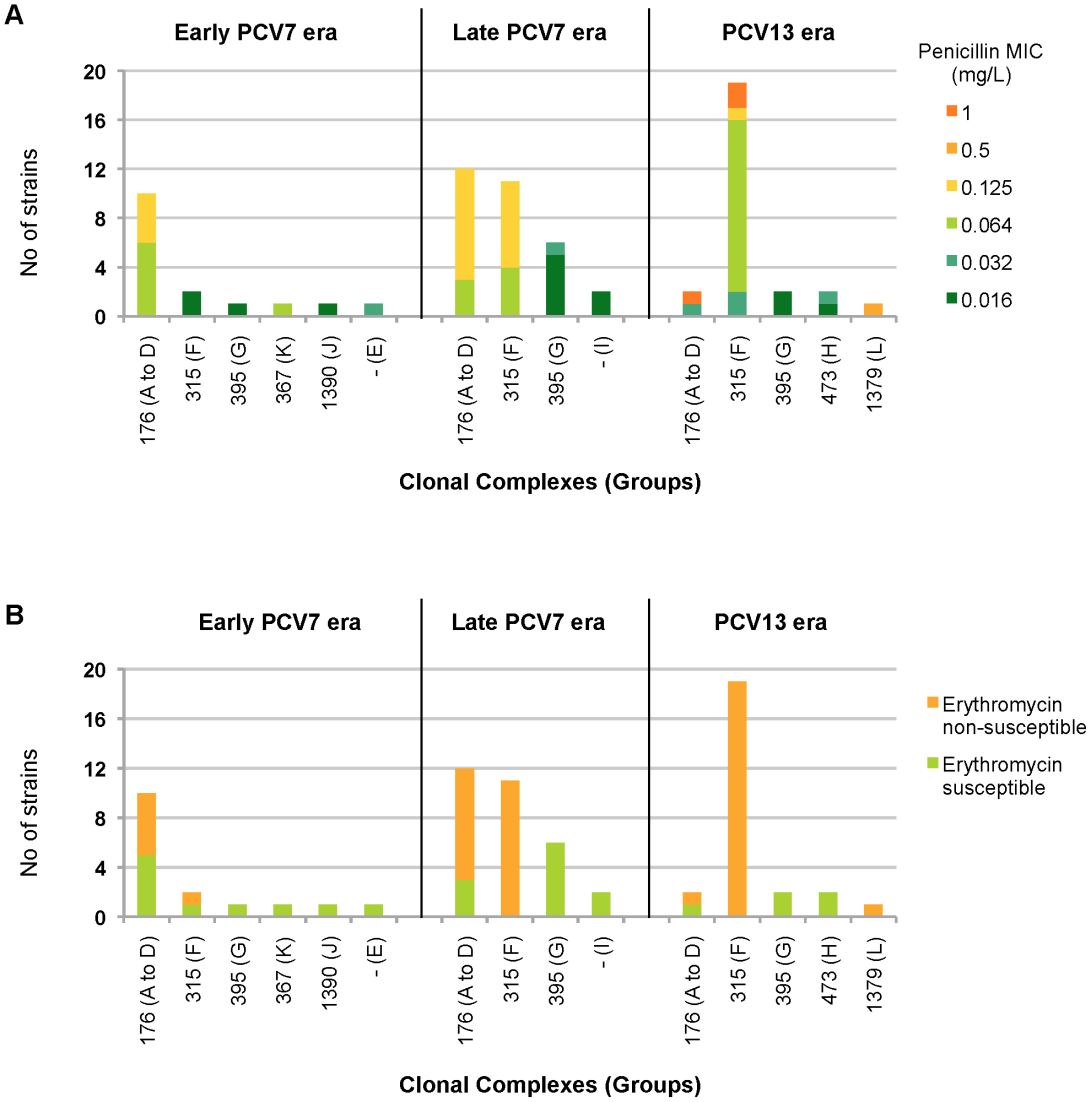
Evolution of clonal complexes of 6C invasive pneumococcal strains according to penicillin (A) or erythromycin (B) susceptibility, during the three study periods (2002–2011). Related groups including STs with at least 5/7 identical alleles (as presented in Table 4) are indicated in brackets.

A total of 101 invasive and carriage isolates were further investigated using MLST (Table 4), to determine whether the increased prevalence in serotype 6C was due to the expansion of a particular clone or to the spread of multiple existing clones. We founded 24 distinct sequence types (STs), 7 of them being new. Three STs were a novel combination of known alleles (STs 8612, 8614, and 8653) and four STs resulted from a known combination including a new allele (*gki* allele 381, *gdh* allele 380, *ddl* allele 556, or *spi* allele 372), resulting in the new STs 8655, 8656, 8657 and 8658, respectively. STs were assigned to 12 groups, one being a singleton. Fifty-three, 28, and seven isolates belonged to three main groups: group A, group F (corresponding to the clonal complex [CC] 315), and group G (corresponding to CC395), respectively. They included 58/71 of IPD and 30/30 of the NP carriage strains, which all belong to group A or F. For both invasive and carriage strains, three STs were predominant, namely ST386 (CC315), ST1150 and ST2267 (both from group A). The evolutionary trend of these STs showed that most of those belonging to group A, and particularly ST1150 and ST2667, tend to disappear after 2009 (Fig. 1). By contrast, ST386 belonging to CC315 dramatically increased from 5% of all STs in the early PCV7 period, to 77% during the PCV13 period (2010–2011).

**Table 4:** Sequence-type (ST) distribution of serotype 6C invasive pneumococcal disease (IPD, n = 71) or nasopharyngeal (NP) colonizing (n = 30) strains.

										No of isolates		
Group*	ST	*aroE*	*gdh*	*gki*	*recP*	*spi*	*xpt*	*ddl*	Putative progenitor°	IPD	NP	Related serotypes°§
**A**	**1150**	7	25	8	6	25	6	8	1150	8	3	**6C**-6A
	**2667**	7	25	8	16	25	6	8		4	4	6C-6A
	**8655**	7	25	381	6	25	6	8		4	0	(6C)
	**2689**	7	25	8	6	25	28	8		0	1	**6C**-(6A)
	**8614**	1	25	8	16	25	6	8		3	0	6C
	**5169**	7	248	8	16	25	6	8		0	1	(NT)
**B**	**8612**	7	5	8	6	6	20	8	–	2	0	6C
**C**	**176**	7	13	8	6	10	6	14	176	1	0	**6B**-6A-6C-(6D)
	**138**	7	5	8	5	10	6	14		1	0	**6B**-6C-(6A-14-18F-23F)
**D**	**8658**	15	17	4	11	372	1	17	–	1	0	(6C)
**E**	**8653**	7	13	1	1	10	6	1	Singleton	1	0	(6C)
**F**	**386**	32	28	1	1	15	52	14	315	30	17	**6C**-6B (NT)
	**315**	20	28	1	1	15	14	14		1	1	**6B**-23F-(3-6A-19A)
	**8656**	32	380	1	1	15	52	14		1	2	(6C)
	**4853**	32	28	1	44	15	52	14		0	1	(6C)
**G**	**1714**	1	5	7	12	17	148	14	395	3	0	**6C**-(6A-23F)
	**1692**	1	5	7	12	17	158	14		3	0	**6C**-6A-(6B)
	**8657**	1	5	7	12	17	158	556		1	0	(6C)
**H**	**1560**	7	25	9	4	15	20	28	473	1	0	(6C)
	**1135**	10	25	78	4	15	20	28		1	0	(6B)
**I**	**1014**	2	10	1	43	6	31	6	–	2	0	(6A-6C-8)
**J**	**1390**	10	13	1	43	98	1	20	1390	1	0	**6A**-**6C** (19A)
**K**	**367**	2	22	4	18	27	4	8	367	1	0	(6C)
**L**	**1379**	1	5	9	12	94	28	20	1379	1	0	**6C**-6A

*STs were grouped on the basis of at least five out of seven identical alleles, according to eBURST analysis;

°According to the Pneumococcal MLST website, http://spneumoniae.mlst.net; 9 Dec 2013, last accessed;

§ In bold, major related serotype; in brackets, single report.

### Trends in Antibiotic Resistance

The overall prevalence of antibiotic resistance to penicillin and erythromycin was higher for serotype 6B pneumococci for both invasive and colonizing strains (Table 5). Neither 6A nor 6C invasive strain resistant to amoxicillin or cefotaxime was isolated from 2002 to 2011 (data not shown). However different trend in penicillin susceptibility were observed in the recent period. Up to 2009, at least 65% of the 6A invasive isolates were susceptible to penicillin (MIC≤0.06 mg/L), but during the 2010–2011 period, penicillin non-susceptible 6A isolates increased, reaching 44%. Conversely, between 2008–2009 and 2010–2011, penicillin non-susceptible 6C invasive isolates decreased significantly from 51% to 23% (p<10^−3^). Within the 67 PNSP 6C strains isolated over the whole study period, penicillin MICs did not exceed 2 mg/L, however 27 strains isolated from CSF overtime had to be considered as penicillin-resistant using the meningitis breakpoints.

**Table 5:** Antibiotic resistance in carriage and invasive 6B, 6A or 6C pneumococcal isolates over time.

Study year	Serotype	Carriage isolates, No (%)			IPD isolates, No (%)						
		Total	Non-susceptibility		Total	Non-susceptibility			Resistance patterns*		
			Pen	E		Pen	E	T	Pen-E	E-T	Pen-E-T
**2002–2003**	6B	71	47 (66.2)	59 (83.1)	199	108 (54.3)	171 (85.9)	85 (42.7)	**60**	45	39
	6A	40	11 (27.5)	12 (30.0)	55	18 (32.7)	28 (50.9)	7 (12.7)	**13**	2	4
	6C	12	7 (58.3)	7 (58.3)	16	4 (25.0)	6 (37.5)	1 (6.3)	3	1	0
**2004–2005**	6B	56	38 (66.7)	48 (85.7)	111	42 (37.8)	88 (79.3)	51 (46.0)	22	**30**	19
	6A	58	14 (24.1)	16 (27.6)	56	19 (33.9)	29 (51.8)	13 (23.2)	**10**	4	8
	6C	22	2 (9.1)	9 (40.9)	16	2 (12.5)	7 (43.8)	4 (25.0)	1	**4**	0
**2006–2007**	6B	21	10 (47.6)	12 (57.2)	72	27 (37.5)	60 (83.3)	35 (48.6)	15	**22**	12
	6A	46	11 (23.9)	14 (30.4)	57	15 (26.3)	17 (29.8)	5 (8.8)	**8**	0	3
	6C	25	11 (44.0)	12 (48.0)	22	10 (45.5)	16 (72.3)	10 (45.5)	4	5	5
**2008–2009**	6B	11	6 (54.6)	7 (63.6)	27	11 (40.7)	21 (77.8)	12 (44.4)	6	**8**	4
	6A	28	6 (21.4)	6 (21.4)	37	11 (29.7)	14 (37.8)	4 (10.8)	**7**	2	1
	6C	45	17 (37.8)	26 (57.8)	59	30 (50.9)	46 (78.0)	38 (64.4)	4	16	**22**
**2010–2011**	6B	11	6 (54.6)	5 (45.5)	12	7 (58.3)	10 (83.3)	8 (66.7)	2	3	4
	6A	14	7 (50.0)	7 (50.0)	32	14 (43.8)	15 (46.9)	6 (18.8)	**6**	3	2
	6C	88	29 (33.0)	67 (76.1)	91	21 (23.1)	71 (78.0)	60 (66.0)	5	**44**	16

Pen, penicillin; E, erythromycin; T, tetracycline.

*The main resistance phenotype is indicated in bold.

The rate of erythromycin resistance increased significantly between 2002–2003 and 2010–2011, from 38% and 58% to 78% and 76% (p<0.01), for invasive and colonizing 6C isolates, respectively (Table 5). A similar trend was observed for tetracycline resistance. By contrast, during the same period, the overall erythromycin resistance of pneumococci isolates from NP carriage sent in our reference center decreased significantly, from 63% in the pre-vaccine era to 43% in the PCV13 era (p<10^−4^) [24], [25].

For 6C isolates, the trends toward increased susceptibility to penicillin on one side, and resistance to erythromycin and tetracycline on the other side, correspond to the clonal expansion of the ST386 clone (Fig. 1).

## Discussion

In France, the IPD incidence significantly decreased in children less than two years from 32.7 to 24.6 cases per 100,000 (−25%, p<10^−4^) between 1998–2002 (pre-vaccine era) and 2008–2009 [26], [27]. This was in contrast with the rise from 8.5 to 10.8 cases per 100,000 (+28%, p<10^−3^) observed in the remaining population. Overtime, the decrease in PCV7 type IPD (−90% in children less than 2 years, −60% in the remaining population) was offset by an increase of non-vaccine type IPD (+138% in children less than 2 years, +110% in the remaining population). It explains why, in France, and in contrast to many countries (11), the overall IPD incidence (children and adults) increased from 9.6 to 10.6 cases per 100,000, between 2001 and 2011, hiding a moderate but significant decrease starting from 2008–2009 (−5%, p = 0.016) (26). In this matter, we investigated the role of the serotype 6C as one of the replacement serotypes in IPD and nasopharyngeal carriage.

Analysis of pneumococci collected at the CNRP since 2002 showed that, as reported worldwide, the introduction of PCV7 was accompanied by an almost disappearance of IPD and NP carriage caused by serotype 6B and, to a lesser extent, by 6A [13]–[18]. For the latter not present in the vaccine, it was suggested to be primary related to a cross protection associated with the PCV7 [12], [28]. During the same period (2003–2011) a serotype replacement within the serogroup 6 was evidenced by a gradual increase of 6C IPD in agreement with the trends observed in USA and in some European countries [14], [15], [29].

We showed that, in France, serotype 6C which was pre-existing to PCV7 introduction (<1% of IPD and NP carriage, Tables 1 and 2) as previously reported [7], [22], became gradually from 2002 to 2011 the most prevalent serotype of serogroup 6 among both invasive and colonizing pneumococci. However, this hides contrasting trends in IPD and NP carriage. The significant increase in 6C IPD observed in adults was not observed in children less than two years, as observed previously by Carvalho *et al*. [14]. By contrast, for this latter population, an increase in 6C carriage was observed. An increase in 6C NP carriage in absence of increase in 6C IPD in children was also reported in other countries [28], [30]. Furthermore, considering the whole study period, the prevalence of 6C in children less than two years was dramatically lower for IPD than for colonizing isolates (7/2,050, 0.3% vs. 192/4,637, 4.0%, p<10^−7^). These observations suggest that serotype 6C could be poorly invasive, mostly causing IPD in the more susceptible and non-vaccinated population, a hypothesis previously proposed by Yildirim *et al*. [31]. Another hypothesis is that the serotype 6B conjugate included in PCV7 could have conferred a partial cross-immunity against 6C, although not evidenced *in vitro* [12], sufficient enough to induce an individual protection against 6C IPD but insufficient to prevent 6C colonization and therefore unable to induce herd-immunity.

Among the 101 invasive and colonizing strains studied by MLST analysis, we found (Table 4) 24 different STs, clustered in 12 groups, reflecting a high genetic diversity previously observed elsewhere [14], [15], [17], [22], [32]. According to the MLST global database, four of the 12 groups (groups A, B, C, and D) fell in group 1, the largest one that results from the merge of two CCs, CC156 and CC176, and that has been recently shown to contain in fact ten genetically distinct evolutionary lineages [33]. Further analysis showed that the nine STs from groups A, B, and C belonged to only two of these lineages, a or b, while ST8658 (group D) was close to ST239, which is assigned to lineage g. These three lineages are however all related to the ancient CC176 [33]. It is noteworthy that no ST was common to STs of strains isolated in United States [14], [15], [32], except for ST1150 [34]. In contrast, we found STs that have been recovered in Europe: ST1692 (CC395) was found mostly in the north of Europe [5], [18], [22], particularly in UK where it was shown to be predominant [35], while ST1150 and its single-locus variants were found to be the dominant STs among colonizing or invasive strains in the south of Europe [17], [36]. This suggests that emergence of serotype 6C isolates is area-dependent, first event of capsular switch occurring in many places of the world between an unknown donor and a pneumococcal strain belonging to one of the major circulating clones.

Most of the STs shared by the 6C strains from our collection were related to 6A and also to 6B serotype in the MLST database (Table 4, http://spneumoniae.mlst.net/sql/burstspadvanced.asp). However, it should be noted that some of these strains may have been only conventionally serotyped, and thus were tested neither for 6C, nor for 6D serotypes.

Over the study period, survey of 6C STs revealed that STs related to CC176 tend to disappear while STs belonging to CC315 have spread (Fig. 1). In addition to the vaccine pressure, it has been proposed that the associated antibiotic resistance traits of 6C would facilitate its emergence. Indeed, a gradual increase in the proportion of PNSP among serotype 6C isolates has been shown to occur in the United States and in Spain [14], [29], [34]. From our results, the increase in erythromycin resistance was associated with the striking expansion of clone ST386 (CC315), which only contains, both in IPD and NP carriage, erythromycin resistant 6C isolates (Table 5, Fig. 1B). To our knowledge, only strains isolated in Denmark and Spain have been previously related to this clone [22], [29]. Among 29/30 IPD isolates belonging to ST386, erythromycin resistance was coupled with tetracycline resistance. This result is not surprising since ST386 is related to the 6B genetic background of the international Poland^6B^-ST315 clone, which harbors the Tn*6002* transposon [29], [37]. We cannot rule out that this clonal expansion was related to the presence of particular loci providing enhanced fitness, as reported by Loman et al. to explain the clonal spread of serotype 6C CC395 in United Kingdom [35].

This study has some limitations: (i) we have not investigated the prevalence of the serotype 6D, but other studies have shown that this serotype is still rare [36], [38], [39]; (ii) data from the pre-vaccine 2002–2003 period collected in this study should be interpreted with caution, taking in account the lack of data during earlier years; (iii) MLST was performed on randomly taken strains, offering a non-exhaustive snapshot of our 6C strains. However, several features make this study unique: (i) the duration of the on-going survey (10 years), (ii) the large scale collection for both IPD and nasopharyngeal colonizing isolates, and (iii) recent data on 6C evolution after implementation of PCV13 in France.

In conclusion, two factors may have contributed to the gradual emergence of a particular successful resistant 6C clone ST386 in France: the vacuum left by 6B and to a lesser extent by 6A isolates under PCV7 pressure along with a lack or poor cross-reaction between 6B conjugate elicited antibodies and 6C strains, and the benefit conferred by the antibiotic resistance under the antibiotic pressure, especially that of macrolides, highly used in France [40].

The use of PCV13, including a 6A conjugate shown to elicit cross-functional opsonophagocytic antibodies against 6C [41], has reduced the risk of 6C nasopharyngeal carriage in vaccinated children in France and Israel (Table 3, [24], [42]). We can therefore expect a significant decrease in 6C IPD in unvaccinated adults due to herd immunity.
